# A Survey of Registered Dietitians’ Concern and Actions Regarding Climate Change in the United States

**DOI:** 10.3389/fnut.2015.00021

**Published:** 2015-07-08

**Authors:** Irana W. Hawkins, Alan L. Balsam, Robert Goldman

**Affiliations:** ^1^Health Professions Education Doctoral Program, Simmons College, Boston, MA, USA; ^2^Brookline Health Department, Brookline, MA, USA; ^3^Department of Mathematics and Statistics, Simmons College, Boston, MA, USA

**Keywords:** climate change, registered dietitians, vegetarian, vegan, practice behaviors

## Abstract

Dietary choices are a tool to reduce greenhouse gas emissions. While registered dietitians are on the front lines of food and nutrition recommendations, it is unclear how many are concerned with climate change and take action in practice in the United States. We explored concern about climate change among registered dietitians, and identified factors that may influence practice-related behaviors. Our study population included a random sample of all registered dietitians credentialed in the United States. Primary data were gathered using a cross-sectional survey. Of the 570 survey responses, 75% strongly agreed or agreed that climate change is an important issue while 34% strongly agreed or agreed that dietitians should play a major role in climate change mitigation strategies. Thirty-eight percent engaged in activities that promoted diet as a climate change mitigation strategy. Vegetarian (*p* = 0.002) and vegan dietitians (*p* = 0.007) were significantly more likely than non-vegetarian and non-vegan dietitians to engage in activities that promoted diet as a climate change mitigation strategy. Overall, concern for climate change among dietitians varied significantly by the region of the country in which the dietitian resided, and awareness that animal products are implicated in climate change. Registered dietitians in the United States are concerned with climate change. However, there is a discrepancy between concern and practice-based actions. These results suggest the need for educational and experiential opportunities connecting climate change mitigation to dietetics practice.

## Introduction

Climate change poses unprecedented global challenges for the living beings and living systems of this planet. The risks to human health, public health, and the natural environment are numerous and include major disruptions to social and economic systems (1–5). Likewise, climate change is an overarching social justice and human rights issue (6, 7). The negative consequences of climate change are projected to increase in severity (8). While greenhouse gas emissions continue to climb, scientists advocate the urgency of reducing emissions along with the associated carbon storage to restore and balance the global climate system – with the additional goal of minimizing irreversible climate change (1, 9, 10).

A burgeoning array of interdisciplinary data implicates food from animal sources in greenhouse gas emissions, increased resource consumption, and environmental degradation (11–26). Ruminants, particularly beef cattle, demonstrate greater environmental burdens than other animals (18, 20, 27, 28). Other studies indicate that plant-based diets and reducing animal product consumption are effective options in ameliorating environmental degradation, decreasing greenhouse gas emissions, and minimizing resource consumption (11–28). Attaining climate mitigation targets may not be possible without reductions in animal product consumption (29, 30). Additionally, the Academy of Nutrition and Dietetics recognizes that well-planned plant-based diets are nutritionally sound throughout the entire lifecycle (31), and are associated with reduced chronic disease morbidity and mortality (25, 32, 33).

There is a growing body of literature regarding concern for climate change among allied health and public health providers. In their survey of United States public health nurses (*n* = 786), Polivka and colleagues found that 75% of respondents agreed that humans are severely abusing the environment (34). Half of the respondents agreed that their nursing department had an obligation to address the health impacts of climate change (34). Most disagreed with the statement that their nursing division was prepared to address the health-related impacts of climate change (34). Truckner surveyed health care providers who were members of the Wilderness Medical Society concerning awareness and beliefs about human-induced environmental degradation (HIED) (35). Of the 658 respondents, 86% were physicians involved in emergency and primary care medicine while one respondent was a registered dietitian (35). Eighty percent of respondents believed that HIED had directly and adversely affected patients, but 93% reported that they do not distribute information about the adverse health effects of HIED to patients (35). A recent survey of African-American physicians with 284 respondents found that while 88% of respondents agree that climate change is directly relevant to patient care, 71% of respondents do not know how to approach the issue with patients (36). Finally, in a survey of local public health department directors in the United States (*n* = 217), 34% report that they have programs to increase the consumption of local, organic, and plant-based foods, while 54% do not have and/or are not planning to address the role of food in climate change mitigation activities (37).

Registered dietitians are well positioned to educate patients, the public, allied healthcare providers, and interdisciplinary colleagues about food choices that can minimize environmental degradation. With a unique skill set that is applied across a variety of settings and institutions, dietitians can take a major leadership role in promoting diet-related climate change mitigation actions while capitalizing on the health benefits of promoting plant-based diets for chronic disease prevention and amelioration (18, 22, 38, 39).

Within the dietetics profession, there has been a growing awareness of the relationship between food systems and impact on the natural environment (40, 41). However, it is unclear how many registered dietitians in the United States are aware of the connection between diet and climate change and take action to mitigate climate change in their professional practice. Thus, the purpose of this research study was to provide a quantitative account of registered dietitians’ beliefs and concerns about climate change; determine registered dietitians’ involvement in climate change mitigation activities; and understand if a relationship exists (if at all) among (a) practice behaviors, (b) concern for climate change, (c) personal dietary behaviors, and (d) demographic variables.

## Materials and Methods

Our study sample consisted of registered dietitians credentialed by the Commission on Dietetic Registration. Along with a viable email address, these were the only inclusion criterion for this study. Our survey was approved by the Academy of Nutrition and Dietetics in January 2012. A cross-sectional survey was created for primary data collection. The survey was tested for face validity and revised accordingly. It contained 52 questions eliciting awareness, beliefs, and concern regarding diet-related climate change and environmental degradation; practice behaviors and outcomes; and personal dietary behaviors. We also captured basic demographic information. Responses included numeric, nominal, ordinal, and four pre-categorized open-ended “other” questions. These “other” questions were scrutinized for validity. Table 1 offers examples of the questions posed.

**Table 1:** **Examples of questions from the survey**.

**I. Questions eliciting concern for climate change**
To what degree do you believe that climate change is an important issue?
□ Strongly agree
□ Agree
□ Unsure
□ Disagree
□ Strongly disagree
To what degree do you believe that climate change is an important practice issue for registered dietitians?
□ Strongly agree
□ Agree
□ Unsure
□ Disagree
□ Strongly disagree
To what degree do you believe that registered dietitians should play a major role in climate change mitigation strategies?
□ Strongly agree
□ Agree
□ Unsure
□ Disagree
□ Strongly disagree
**II. Questions eliciting practice behaviors**
Do you engage in activities that promote diet as a climate change mitigation strategy?
□ Yes
□ No
Do you recommend organic foods in practice (when at work)?
□ Yes
□ No
Do you recommend locally produced foods in practice (when at work)?
□ Yes
□ No
**III. Questions eliciting personal behaviors**
Do you obtain food from farmers markets, community supported agriculture, and/or other locally grown sources?
□ Yes
□ No
Are you vegan (avoid the consumption of all animal products)?
□ Yes
□ No
Are you vegetarian (avoid the consumption of animal flesh including fish but may include eggs, dairy products, etc.)
□ Yes
□ No

We investigated whether being vegetarian or vegan impacted actions to mitigate climate change due to presumed level of comfort with plant-based diets. Hence, several hypotheses were proposed at the outset of the study including: (1) The personal dietary behavior of being either vegetarian or vegan predicts the action of promoting diet as a climate change mitigation strategy among registered dietitians, (2) the belief that animal products are not *essential* for a healthy diet predicts the action of promoting diet as a climate change mitigation strategy among registered dietitians, and (3) comfort in promoting solely plant-based (vegan) diets predicts the action of promoting diet as a climate change mitigation strategy among registered dietitians.

A base target sample size of 383 was required from the total universe of 84,146 registered dietitians in the United States using a 5% margin of error, a 95% confidence level, and an anticipated response rate of 20%. Thus, we invited 1,915 dietitians to participate in the study. The technology team at the Academy of Nutrition and Dietetics provided the random sample from their current database of registered dietitians credentialed in the United States. The survey was completed online via the Internet-based SurveyMonkey (Palo Alto, CA, USA) research service.

An email including an explanation of the study, informed consent, and a link to the survey was sent on March 7, 2012. The ability to enter a drawing for a $100 gift card was offered to thank potential study participants for their time and efforts. Because the response rate was less than anticipated on March 21, 2012 (there were 305 responses, short of the target of *n* = 383), the Academy of Nutrition and Dietetics provided another random sample of 1,994 registered dietitians. The second round started on March 22, 2012 and continued for 1 week until midnight March 29, 2012. Over the two rounds, a total of 625 registered dietitians provided consent.

Approximately, 91 email addresses were returned with error messages. For data integrity purposes, duplicate IP addresses were checked against the original subject lists. Three survey responses were eliminated because those individuals were not included in the original sample. Those individuals who provided consent but did not otherwise complete any part of the survey were also eliminated.

The survey data were analyzed using SPSS version 20.0 (IBM Corporation, Armonk, NY, USA). Descriptive statistics and inferential statistics including the chi-square test for independence, the chi-square goodness of fit test, Fisher’s exact test, and logistic regression were used. There was limited knowledge of how the study participants (n = 570) differed (if at all) from those dietitians in the entire study population (3,909 potential survey participants). Therefore, the chi-square goodness of fit test was utilized to check the extent to which the distribution of the variable region (the only variable available among both groups) differed at all between all study participants and all potential survey participants. In fact, dietitians in the South participated in the study less often than expected, while more dietitians participated from the West and Midwest than anticipated, respectively. The chi-square goodness of fit test did reveal a statistically significant difference between the two groups, p = 0.0136. Therefore, it was important to further understand if this difference impacted the overall results.

The percentage of respondents holding particular views were calculated using the “population” weights (the percentage of respondents in each region in the population) rather than the sample weights (the percentage of respondents in each region within the sample). In each case, there was very little difference in the percentages. For example, 74.9% of respondents either strongly agree or agree that climate change is an important issue. When the population “weights” (proportion of the population in each region) were used, the corresponding percentage is 74.1. Similar results were obtained with other responses to other questions. This all suggests that differences between the sample and the study population are unlikely to affect the conclusions of this study.

Survey participants were free to answer the questions as desired, and thus, every question was optional as indicated in the consent form. Not all survey participants answered every question. All data analyses were based only on non-missing data. Statistical significance was achieved if the *p*-value was <0.05.

## Results

The response rate was 14.6% with n = 570 usable responses from 3,909 potential survey participants. Approximately 96% of survey respondents were women. Survey participants resided in all 50 states – but not the District of Columbia. One survey participant resided in Guam, while another resided in Canada. Over 92% of study participants describe themselves as White/Caucasian, followed by Asian (5%), Black/African-American (2%), American-Indian or Alaska Native (0.4%), Other/Mixed Race (0.4%), and Native Hawaiian or Other Pacific Islander (0.2%), respectively. Two percent of survey participants described their ethnicity as being Hispanic or Latino. Survey participants ranged in age from 23 to 75 years, with the mean being 42.7 years and the SD, 12.3 years. The number of years in dietetics practice ranged from 1 to 49, with a mean of 16 years and an SD of 11.6 years. The number of years of higher education obtained ranged from 4 to 20, with a mean of 5.8 years and an SD, 2.6 years. The majority of survey participants represented the practice area of clinical dietetics (55.0%), followed by Public Health (16.0%) and Education/Teaching and Research (8%).

### Concern for climate change

Approximately, 75% of survey participants either strongly agree or agree that climate change is an important issue. Over 45% of survey participants either strongly agree or agree that climate change is an important practice issue for registered dietitians, while almost 50% are unsure that registered dietitians should play a *major role* in climate change mitigation strategies. However, 34% strongly agree or agree that registered dietitians should play a major role in climate change mitigation strategies.

### Predicting belief that climate change is an important issue

Table 2 shows the results of a logistic regression analysis predicting the probability of the belief (strongly agree, agree) that climate change is an important issue based on demographic variables. For the predictor variable region of the country, the South was the reference region. The corresponding *p*-values of the indicator variables, West (*p* = 0.003), Midwest (*p* = 0.008), and Northeast (*p* = 0.00005) denote that the effects of these variables are statistically significant. That is, if living in the Midwest, West, or Northeast, the odds of strongly agreeing or agreeing that climate change is an important issue is 2.1, 2.4, and 3.5 times that of those living in the South (the reference region), respectively – holding all other variables constant.

**Table 2:** **Logistic regression analyses predicting strong agreement or agreement that climate change is an important issue based on demographic variables**.

Predictor variable	*p*-Value	Odds ratio
West	0.003	2.442
Midwest	0.008	2.128
Northeast	0.00005	3.537
Gender	0.922	0.943
White/Caucasian	0.265	0.581
Ethnicity	0.178	0.236
Area of practice – clinical nutrition	0.172	0.692
Area of practice – public health	0.095	0.560
Age	0.465	1.015
Years of practice	0.053	0.960
Years of education	0.704	0.982
Constant	0.044	17.864

### Actions related to climate change mitigation

Approximately, 38% of respondents engage in activities that promote diet as a climate change mitigation strategy. Eight percent of workplaces provided funding for diet-related climate change mitigation activities. A total of 194 survey participants (34%) reported activities that promote diet as a climate change mitigation strategy. In particular, one respondent reported influencing institutional policy changes.

### The relationship between beliefs, personal dietary behaviors, and action

Dietitians were divided when asked if animal products are *essential* for a healthy diet. Nearly 42% of survey participants either strongly disagreed or disagreed that animal products are essential for a healthy diet, while 8% are unsure. Almost 50% of survey participants either strongly agreed or agreed that animal products are essential for a healthy diet.

Next, we tested the hypothesis that the variable personal dietary behavior of whether or not vegetarian or whether or not vegan predicts the promotion of diet as a climate change mitigation strategy among registered dietitians. Of note is that more than 10% of survey participants reported being vegetarian, while 2% reported being vegan. Tables 3 and 4 show the analysis of the survey participants that engage in activities that promote diet as a climate change mitigation strategy based on the personal behaviors of being vegetarian or vegan, respectively. The corresponding *p*-value for both the chi-square test for independence (0.002) and the Fisher’s exact test (0.007) are exceedingly small, indicating that the results are statistically significant. These data suggest that vegetarian and vegan dietitians are significantly more likely than non-vegetarian and non-vegan dietitians to engage in activities that promote diet as a climate change mitigation strategy.

**Table 3:** **Number and percentage of survey participants that engage in activities that promote diet as a climate change mitigation strategy based upon being vegetarian^a,b,c^**.

	Vegetarian (yes) (*n* = 53)	Vegetarian (no) (*n* = 341)
Number and percentage that engage in activities that promote diet as a climate change mitigation strategy	30 (56.6%)^d^	162 (34.6%)

*^a^Difference = 22%; ^b^95% CI = 7.0–36%; ^c^Pearson chi-square = 9.9; ^d^p-value <0.002*.

**Table 4:** **Number and percentage of survey participants that engage in activities that promote diet as a climate change mitigation strategy based upon being vegan^a,b^**.

	Vegan (yes) (*n* = 10)	Vegetarian (no) (*n* = 512)
Number and percentage that engage in activities that promote diet as a climate change mitigation strategy	8 (80%)^c^	186 (36.3%)

*^a^Difference = 43.7%; ^b^95% CI = 18.5–68.8%; ^c^Fisher’s exact test, p-value <0.007*.

Following this, we tested the hypothesis that the belief that animal products *are not essential for a healthy diet* predicts the promotion of diet as a climate change mitigation strategy. The corresponding *p*-value (0.013) for the number and percentage of survey participants that engage in activities that promote diet as a climate change mitigation strategy based on the belief that animal products are not essential for a healthy diet is exceedingly small, indicating that the results are statistically significant. These data suggest that those who believe that animal products are not essential for a healthy diet are significantly more likely to engage in activities that promote diet as a climate change mitigation strategy than those who do believe that animal products are essential for a healthy diet.

Lastly, we tested the hypothesis that self-efficacy (comfortable, very comfortable) in promoting solely plant-based (vegan) diets predicts involvement in diet and climate change mitigation strategies. Table 5 shows the analysis.

**Table 5:** **Number and percentage of survey participants that engage in activities that promote diet as a climate change mitigation strategy based on self-efficacy (comfortable, very comfortable) in promoting a plant-based diet^a,b,c^**.

	Self-efficacy (yes) (*n* = 193)	Self-efficacy (no) (*n* = 330)
Number and percentage that engage in activities that promote diet as a climate change mitigation strategy	93 (48%)^d^	101 (30.6%)

*^a^Difference = 17.6%; ^b^95% CI = 8.9–26.2%; ^c^Pearson chi-square = 16.1; ^d^p-value < 0.00005*.

The corresponding *p*-value (0.00005) is exceedingly small and <0.05, indicating that the results are statistically significant. These data suggest that those who report self-efficacy in promoting plant-based (vegan) diets are significantly more likely to engage in activities that promote diet as a climate change mitigation strategy than those who report that they are less comfortable in promoting plant-based diets.

## Discussion

To our knowledge, this is the first study to collectively understand concerns and actions to mitigate climate change among a random sample of all registered dietitians in the United States. This study yielded several important findings: (1) the majority of registered dietitians surveyed agreed that climate change is an important issue, but are largely unsure that registered dietitians should play a major role in climate change mitigation strategies; (2) the region of the country that one resides is the demographic variable that was an important predictor of the belief that climate change is an important issue; (3) being vegetarian or vegan was significantly associated with actions promoting diet as a climate change mitigation strategy; (4) the belief that animal products are not essential in the diet was significantly associated with actions promoting diet as a climate change mitigation strategy; (5) comfort in promoting solely plant-based diets was significantly associated with actions that promote diet as a climate change strategy; and (6) the gap between concern for climate change and practice behaviors suggests a lack of knowledge or self-efficacy in connecting practice behaviors to climate change mitigation.

In an era where scientists forecast the current and future dimensions of climate change as worrisome, it is reassuring that 75% of registered dietitians agree or strongly agree that climate change is an important issue. This is consistent with the majority of Americans who believe in the reality of global warming (42). However, only 46% of registered dietitians strongly agreed or agreed that climate change is an important practice issue and 50% are unsure if registered dietitians should play a major role in climate change mitigation strategies. This is similar to the findings of Sulda et al. in their survey of South Australian dietitians and nutritionists who ranked concern for climate change at a mean of 8.5 out of 10 with 10 being extremely important – but then ranked it lower in importance to a dietitian’s professional work in overweight and obesity, food security, diabetes, etc. (43). Dietary recommendations to ameliorate nutrition-related chronic diseases can offer simultaneous co-benefits to the natural environment, such as reduced greenhouse emissions, eliminating the notion of competing interests (18, 25, 38, 39). Thus, offering dietitians educational opportunities and experiences that increase knowledge, skills, and abilities with regard to diet-related climate change mitigation as well as the resultant co-benefits could prove to be highly beneficial.

Approximately 38% of survey participants engage in activities that promote diet as a climate change mitigation strategy. This corresponds with the 34% that strongly agreed or agreed that registered dietitians should play a major role in climate change mitigation strategies and the 45% that strongly agree or agree that climate change is an important practice-based issue. While not the majority, it is still impressive considering that only 8% reported that their respective workplace provided funding for diet and climate change mitigation activities. Nevertheless, the substantial gap between the 75% that strongly agree or agree that climate change is an important issue and the 38% that promote diet as a climate change mitigation strategy should be noted. It may be that dietitians lack the necessary education, experiences, or skills to take action (44, 45). Or, like the general population, it may be that climate change is seen as a distant threat (42). Wilson and Garcia found that clinical dietitians in Canada do not routinely discuss the impact of food choices on the natural environmental with patients (46). In recent years though, some dietetic internship programs in the United States have incorporated “Sustainable Food Systems” as an emphasis in their accredited training program (44), while some nutrition degrees from the associates level (47) through the graduate level (48) and also offer such programs that even span across departments (48). It will be important to learn the effect of these training programs on future dietetics-related practice behaviors and the associated environmental outcomes. Additionally, exposing students to risk-taking and diplomacy may prove fruitful (49). Similar to other studies of allied health and public health providers (34–37), our results demonstrate that there is a need within and across health disciplines for skill in incorporating climate change mitigation into practice.

Region of the country in which a registered dietitian resided was an important predictor in strongly agreeing or agreeing that climate change is an important issue. Dietitians residing in the South were significantly less likely to strongly agree or agree that climate change is an important issue. Certainly, norms, beliefs, and attitudes may exist and persist depending on the state and region in which one resides. Moreover, media coverage of diet-related climate change issues varies in certain geographic regions, and has been under-represented altogether in the media in previous years – particularly in the South (50). Because of the urgent nature of climate change, further investigation of what norms enhance or detract from the importance of climate change mitigation in practice is warranted – including those that occur from within the profession itself. Qualitative research may be particularly helpful in this instance. Other useful research may include the number of dietitians employed by the livestock industry in the South compared to other regions of the country; understanding exposure (or lack thereof) to diet-related climate change mitigation in training experiences and didactic education; and even understanding attitudes about energy sector jobs in the South.

Although our level of statistical significance was articulated at the outset of the study (*p* = 0.050), our logistic regression analysis found that the variable “Number of Years in Practice” approached statistical significance at 0.053. After holding all other variables constant, the odds of agreeing that climate change is an important issue negligibly declined with increasing number of years in practice. Further research is warranted to understand this trend. However, it is plausible that because climate change is a recently documented phenomenon, dietitians practicing longer may have spent less time being aware of climate change than more recently trained dietitians.

Some practice-based publications have called upon registered dietitians to take action in their professional practice and personal lives to minimize the impact on the natural environment, including increasing plant-based protein consumption (40, 41). Interestingly, we found a significant relationship between the personal behaviors of being vegan or vegetarian and practice outcomes, as well as comfort in promoting solely plant-based diets. These findings have important implications for dietetics education, public health practice, and continuing education. Providing knowledge, skills, and experiences to increase skill and comfort in promoting plant-based meals among registered dietitians could make a difference with regard to practice-based recommendations. Dietitians in turn can share such skills across the healthcare, agriculture, food systems, environmental health, and the public health spectrum (3, 18, 51). It is important also to note that dietitians were nearly divided with regard to the statement that animal products are *essential* for a healthy diet. Thus, understanding dietitians’ beliefs about the role of animal products and plant-based foods may be key to understanding practice-based actions to reduce impact on the natural environment.

Just over 10 percent (10.3%) of survey participants reported being vegetarian, while 2% reported being vegan. It has been estimated that over 2% of the American population is vegetarian, and just over 2% is vegan (52). Dietitians participating in this survey reported being vegetarian in higher percentages than the general population, while those that reported being vegan is consistent with the general population.

Additionally, it is important to point out that although general messages to eat less animal products to mitigate climate change are appropriate and constructive, substantive recommendations, and meal planning advice are also warranted. Registered dietitians proficient in plant-based dietary patterns can offer suggestions across the entire lifecycle, disease conditions, and food planning and food procurement settings. For instance, non-governmental environmental organizations that are uncomfortable offering dietary advice as a way to reduce impact on the natural environment (53) may find great benefit in partnering with registered dietitians.

This may be the first study to examine concerns and actions regarding climate change among a randomly selected sample of all registered dietitians credentialed in the United States. It may also be the first to quantitatively examine the relationship between the personal behaviors with regard to climate change mitigation efforts. However, this study did not query those who intentionally restrict their respective intake of animal products who may be considered semi-vegetarian or “flexetarian,” which is worthy of future study. Because this was an exploratory study, further inquiry over time is necessary. It is important to note that those who completed the online survey may be different than those who did not complete the online survey. That is, they could have been a self-selected group that is more likely to be supportive of the topic than those that did not respond. Our response rate was low, and we do not know if all intended survey recipients received our email, as filters may have prevented our survey from reaching the respective dietitian’s email inbox. Furthermore, it could be that those who participated in the survey provided responses based on the nature of questions, termed social desirability bias (54). Lastly, further testing the reliability of our survey tool is important for future research.

Nutrition professionals are called upon to help improve planetary health. Because of their skill set and their unique role in healthcare and in all aspects of food and nutrition – registered dietitians are well positioned to become increasingly important allies in novel approaches to climate change mitigation strategies. Efforts that enable more registered dietitians to build knowledge, skills, and self-efficacy regarding diet-related climate change mitigation activities that they utilize in practice could substantially improve both public health and planetary health.

## Author Contributions

All authors substantially contributed to the conception and design of the study. IH facilitated the research and analyzed the data/performed statistical analysis with the exception of the chi-square goodness of fit test and the population weight distributions that RG performed. RG oversaw data analysis. All authors substantially contributed to the interpretation of the data. IH crafted the manuscript, while AB and RG offered critical insight.

## Conflict of Interest Statement

The authors declare that the research was conducted in the absence of any commercial or financial relationships that could be construed as a potential conflict of interest.

## References

[1] Hansen J Kharecha P Sato M Masson-Delmotte V Ackerman F Beerling DJ Assessing “dangerous climate change”: required reduction of carbon emissions to protect young people, future generations and nature. PLoS One (2013) 12:e81648. 10.1371/journal.pone.0081648 24312568 PMC3849278

[2] Field CB Barros VR Dokken DJ Mach KJ Mastrandrea MD Bilir TE editors. Climate Change 2014: Impacts, Adaptation, and Vulnerability. Part A: Global and Sectoral Aspects. Contribution of Working Group II to the Fifth Assessment Report of the Intergovernmental Panel on Climate Change. New York, NY: Cambridge University Press (2014).

[3] Patz JA Frumkin H Holloway T Vimont DJ Haines A. Climate change challenges and opportunities for global health. JAMA (2014) 312:1565–80. 10.1001/jama.2014.13186 25244362 PMC6108836

[4] McMichael AJ Globalization, climate change, and human health. N Engl J Med (2013) 368:1335–43. 10.1056/NEJMra1109341 23550671

[5] Solomon S Plattner GK Knutti R Friedlingstein P. Irreversible climate change due to carbon dioxide emissions. Proc Natl Acad Sci U S A (2009) 106:1704–9. 10.1073/pnas.0812721106 19179281 PMC2632717

[6] Adger WN Barnett J Chapin FS Ellemor H This must be the place: underrepresentation of identity and meaning in climate change decision-making. Global Environ Polit (2011) 11:1–25. 10.1162/GLEP_a_00051

[7] United Nations. Millennium Development Goals Report 2010. New York, NY: United Nations (2010).

[8] Melillo JM Richmond TC Yohe GW, editors. Climate Change Impacts in the United States: The Third National Climate Assessment. Washington, DC: U.S. Global Change Research Program (2014). 841 p.

[9] Friedlingstein P Andrew RM Rogelj J Peters GP Canadell JG Knutti R Persistent growth of CO_2_ emissions and implications for reaching climate targets. Nat Geosci (2014) 7:709–15. 10.1038/ngeo2248

[10] Le Quéré C Moriarty R Andrew RM Peters GP Ciais P Friedlingstein P Global carbon budget 2014. Earth Syst Sci Data Discuss (2014) 7:521–610. 10.5194/essdd-7-521-2014

[11] Mekonnen MM Hoekstra AY A global assessment of the water footprint of farm animal products. Ecosystems (2012) 15:401–15. 10.1007/s10021-011-9517-8

[12] The Pew Charitable Trusts and Johns Hopkins Bloomberg School of Public Health. Putting Meat on the Table: Industrial Farm Animal Production in America. Washington, DC: Pew Commission on Industrial Farm Animal Production (2008).

[13] Wecht KJ Jacob DJ Frankenberg C Jiang Z Blake DR Mapping of North American methane emissions with high spatial resolution by inversion of SCIAMACHY satellite data. J Geophys Res Atmos (2014) 119:7741–56. 10.1002/2014JD021551

[14] Steinfeld H Gerber P Wassenaar R Castel V Rosales M deHaan C Livestock’s Long Shadow: Environmental Issues and Options. Rome: Food and Agriculture Organization of the United Nations (2006).

[15] Stehfast E Bouwman L vanVuuren DP Climate benefits of changing diet. Clim Change (2009) 95:83–102. 10.1007/s10584-008-9534-6

[16] Foley JA Ramankutty N Brauman KA Cassidy ES Gerber JS Johnston M Solutions for a cultivated planet. Nature (2011) 478:337–42. 10.1038/nature10452 21993620

[17] Marlow HJ Hayes WK Soret S Carter RL Schwab ER Sabaté J. Diet and the environment: does what you eat matter?. Am J Clin Nutr (2009) 89:1699S–703S. 10.3945/ajcn.2009.26736Z 19339399

[18] Tilman D Clark M. Global diets link environmental sustainability and human health. Nature (2014) 515:518–22. 10.1038/nature13959 25383533

[19] Pelletier N Tyedmers P. Forecasting potential global environmental costs of livestock production 2000-2050. Proc Natl Acad Sci U S A (2010) 107:18371–4. 10.1073/pnas.1004659107 20921375 PMC2972962

[20] Masset G Soler LG Vieux F Darmon N. Identifying sustainable foods: the relationship between environmental impact, nutritional quality, and prices of foods representative of the French diet. J Acad Nutr Diet (2014) 114:862–9. 10.1016/j.jand.2014.02.002 24703928

[21] González AD Frostell B Carlsson-Kanyama A Protein efficiency per unit energy and per unit greenhouse gas emissions: potential contribution of diet choices to climate change mitigation. Food Policy (2011) 36:562–70. 10.1016/j.foodpol.2011.07.003

[22] Westhoek H Lesschen JP Trudy Rood T Wagnera S De Marco A Murphy-Bokern D Food choices, health, and environment: effects of cutting Europe’s meat and dairy intake. Global Environ Change (2014) 26:196–205. 10.1016/j.gloenvcha.2014.02.004

[23] Soret S Mejia A Batech M Jaceldo-Siegl K Harwatt H Sabaté J. Climate change mitigation and health effects of varied dietary patterns in real-life settings throughout North America. Am J Clin Nutr (2014) 100:476S–82S. 10.3945/ajcn.113.071589 24898230

[24] Miller SM Wofsky SC Michalak AM Kort EA Andrews AE Biraud SC Anthropogenic emissions of methane in the United States. Proc Natl Acad Sci U S A (2013) 110:20018–22. 24277804 10.1073/pnas.1314392110 PMC3864315

[25] Scarborough P Appleby PN Mizdrak A Briggs AD Travis RC Bradbury KE Dietary greenhouse gas emissions of meat-eaters, fish-eaters, vegetarians and vegans in the UK. Clim Change (2014) 125:179–92. 10.1007/s10584-014-1169-1 25834298 PMC4372775

[26] Weber CL Matthews HS. Food-miles and the relative climate impacts of food choices in the United States. Environ Sci Technol (2008) 42:3508–13. 10.1021/es702969f 18546681

[27] Eshel G Shepon A Makov T Milo R. Land, irrigation water, greenhouse gas, and reactive nitrogen burdens of meat, eggs, and dairy production in the United States. Proc Natl Acad Sci U S A (2014) 111(33):11996–2001. 10.1073/pnas.1402183111 25049416 PMC4143028

[28] Gerber PJ Steinfeld H Henderson B Mottet A Opio C Dijkman J Tackling Climate Change Through Livestock – A Global Assessment of Emissions and Mitigation Opportunities. Rome: Food and Agriculture Organization of the United Nations (FAO) (2013).

[29] Hedenus F Wirsenius S Johansson D The importance of reduced meat and dairy consumption for meeting stringent climate change targets. Clim Change (2014) 124:79–91. 10.1007/s10584-014-1104-5

[30] Davidson EA Representative concentration pathways and mitigation scenarios for nitrous oxide. Environ Res Lett (2012) 7:024005 10.1088/1748-9326/7/2/024005

[31] Craig WJ Mangels AR. Position of the American Dietetic Association: vegetarian diets. J Am Diet Assoc (2009) 109:1266–82. 10.1016/j.jada.2009.05.027 19562864

[32] Key TJ Appleby PN Crowe FL Bradbury KE Schmidt JA Travis RC. Cancer in British vegetarians: updated analyses of 4998 incident cancers in a cohort of 32,491 meat eaters, 8612 fish eaters, 18,298 vegetarians, and 2246 vegans. Am J Clin Nutr (2014) 100(Suppl 1):378S–85S. 10.3945/ajcn.113.071266 24898235 PMC4144109

[33] Orlich MJ Fraser GE. Vegetarian diets in the Adventist Health Study 2: a review of initial published findings. Am J Clin Nutr (2014) 100(Suppl 1):353S–8S. 10.3945/ajcn.113.071233 24898223 PMC4144107

[34] Polivka BJ Chaudry RV Crawford JM. Public health nurses’ knowledge and attitudes regarding climate change. Environ Health Perspect (2012) 120(3):321–5. 10.1289/ehp.1104025 22128069 PMC3295355

[35] Truckner RT. Health care provider beliefs concerning the adverse health effects of environmental and ecosystem degradation. Wilderness Environ Med (2009) 20(3):199–211. 10.1580/08-WEME-OR-222R1.1 19737040

[36] Sarfaty M Mitchell M Bloodhart B Maibach EW. A survey of African American physicians on the health effects of climate change. Int J Environ Res Public Health (2014) 11:12473–85. 10.3390/ijerph111212473 25464138 PMC4276625

[37] Maibach EW Chadwick A McBride D Chuk M Ebi KL Balbus J. Climate change and local public health in the United States: preparedness, programs and perceptions of local public health department directors. PLoS One (2008) 3(7):e2838. 10.1371/journal.pone.0002838 18665266 PMC2474970

[38] Friel S Dangour AD Garnett T Lock K Chalabi Z Roberts I Public health benefits of strategies to reduce greenhouse-gas emissions: food and agriculture. Lancet (2009) 374:2016–25. 10.1016/S0140-6736(09)61753-0 19942280

[39] Haines A Smith KR Anderson D Epstein PR McMichael AJ Roberts I Policies for accelerating access to clean energy, improving health, advancing development, and mitigating climate change. Lancet (2007) 370:1264–81. 10.1016/S0140-6736(07)61257-4 17868819

[40] Harmon AH Gerald BL. Position of the American Dietetic Association: food and nutrition professionals can implement practices to conserve natural resources and support ecological sustainability. J Am Diet Assoc (2007) 107:1033–43. 10.1016/j.jada.2007.04.018 17571455

[41] Tagtow A Robien K Bergquist E Bruening M Dierks L Hartman BE Academy of Nutrition and Dietetics: standards of professional performance for registered dietitian nutritionists (competent, proficient, and expert) in sustainable, resilient, and healthy food and water systems. J Acad Nutr Diet (2014) 114:475–88. 10.1016/j.jand.2013.11.011 24534371

[42] Leiserowitz A Maibach E Roser-Renouf C Feinberg G Howe P Climate Change in the American Mind: Americans’ Global Warming Beliefs and Attitudes in September, 2012. New Haven, CT: Yale Project on Climate Change (2012).

[43] Sulda H Coveney J Bentley M. An investigation of the ways in which public health nutrition policy and practices can address climate change. Public Health Nutr (2010) 13:304–13. 10.1017/S1368980009990334 19545472

[44] Webber CB Sarjahani A Fitting sustainable food systems into dietetic internships-A growing trend. J Hunger Environ Nutr (2011) 6:477–89. 10.1080/19320248.2011.627304

[45] Robinson R Smith C. Integrating issues of sustainably produced foods into nutrition practice: a survey of Minnesota Dietetic Association members. J Am Diet Assoc (2003) 103:608–11. 10.1053/jada.2003.50113 12728221

[46] Wilson ED Garcia AC. Environmentally friendly health care food services: a survey of beliefs, behaviours, and attitudes. Can J Diet Pract Res (2011) 72:117–22. 10.3148/72.3.2011.117 21896245

[47] Mesa Community College. Associate in Applied Science in Sustainable Food Systems [Internet]. (0000). Available from: http://www.mesacc.edu/departments/exercise-science/food-nutrition/sustainable-food-systems/aas-sustainable-food-systems

[48] Montana State University. Sustainable Food and Bioenergy Systems Major-Sustainable Food Systems Option. [Internet]. (0000). Available from: http://www.montana.edu/hhd/undergrad/foodandnutrition/sustainablefood.html

[49] Hawkins IW Balsam A Graves D A qualitative study of the skills that enabled the pro-environmental behaviors of registered dietitians. J Hunger Environ Nutr (2015) 10(1):60–71. 10.1080/19320248.2014.929546

[50] Neff RA Chan IL Smith KG. Yesterday’s dinner, tomorrow’s weather, today’s news? US newspaper coverage of food system contributions to climate change. Public Health Nutr (2008) 12:1006–14. 10.1017/S1368980008003480 18702838

[51] Horton R Beaglehole R Bonita R Raeburn J McKee M Wall S From public to planetary health: a manifesto. Lancet (2014) 383:847 10.1016/S0140-6736(14)60409-8 24607088

[52] Stahler C How often do Americans eat vegetarian meals? And how many adults in the U.S. are vegan? Veg J (2011) 30(4):10–1.

[53] Laestadius LI Neff RA Barry CL Frattarolib S “We don’t tell people what to do”: an examination of the factors influencing NGO decisions to campaign for reduced meat consumption in light of climate change. Global Environ Change (2014) 29:32–40. 10.1016/j.gloenvcha.2014.08.001

[54] van de Mortel T. Faking it: social desirability response bias in self report research. Aust J Adv Nurs (2008) 25:40–8.11480802

